# Obesity, but Not Overweight, Is Associated with Increased Presepsin Levels in Infection-Free Individuals: An Exploratory Study

**DOI:** 10.3390/biomedicines13030701

**Published:** 2025-03-12

**Authors:** Theocharis Koufakis, Dimitrios Kouroupis, Georgios Dimakopoulos, Theofylaktos Georgiadis, Areti Kourti, Panagiotis Doukelis, Ioanna Zografou, Dimitrios Patoulias, Djordje S. Popovic, Athina Pyrpasopoulou, Luca Busetto, Alexander Kokkinos, Vasilios Tsimihodimos, Kalliopi Kotsa, Michael Doumas, Kali Makedou

**Affiliations:** 1 Second Propaedeutic Department of Internal Medicine, Hippokration General Hospital, Aristotle University of Thessaloniki, 54642 Thessaloniki, Greece; dimcour841@gmail.com (D.K.); pitdukel@yahoo.gr (P.D.); ioannazo@yahoo.gr (I.Z.); dipatoulias@gmail.com (D.P.); a.pyrpasopoulou@doctors.org.uk (A.P.); doumasm@auth.gr (M.D.); 2 BIOSTATS, Epirus Science and Technology Park Campus, University of Ioannina, 45110 Ioannina, Greece; info@biostats.gr; 3 National Primary Health Care Network, 61200 Polykastron, Greece; gfiltheo@hotmail.com; 4 Laboratory of Biochemistry, AHEPA University Hospital, School of Medicine, Aristotle University of Thessaloniki, 54124 Thessaloniki, Greecekmakedou@auth.gr (K.M.); 5 Clinic for Endocrinology, Diabetes and Metabolic Disorders, Clinical Centre of Vojvodina, Medical Faculty, University of Novi Sad, 21000 Novi Sad, Serbia; djordje.popovic@mf.uns.ac.rs; 6 Department of Medicine, University of Padova, 35128 Padova, Italy; luca.busetto@unipd.it; 7 First Department of Propaedeutic Internal Medicine and Diabetes Center, Medical School, Laiko General Hospital, National and Kapodistrian University of Athens, 11527 Athens, Greece; akokkinos@med.uoa.gr; 8 Department of Internal Medicine, University of Ioannina, 45500 Ioannina, Greece; vtsimi@uoi.gr; 9 Division of Endocrinology and Metabolism and Diabetes Center, First Department of Internal Medicine, AHEPA University Hospital, Medical School, Aristotle University of Thessaloniki, 54636 Thessaloniki, Greece; kkalli@auth.gr

**Keywords:** presepsin, obesity, overweight, inflammation, biomarker

## Abstract

**Background/Objectives**: Intestinal dysbiosis and systemic inflammation are involved in the pathophysiology of obesity and its complications. Presepsin is a recently discovered inflammation marker, being the soluble form of the bacterial lipopolysaccharide (LPS) receptor. Due to the imbalance of the gut flora and subsequent disruption of the intestinal barrier, circulating LPS levels have been found to be elevated in patients with metabolic diseases, even in the absence of infection. However, to date, no studies have evaluated whether obesity is associated with elevated presepsin levels. **Methods**: The present study included 81 participants (61.7% women, 27 with obesity, 34 with overweight, and 20 controls with normal body mass index), all free of infection and diabetes mellitus. Presepsin was measured in serum by ELISA, and its concentrations were compared between the groups. **Results**: The obesity group had higher presepsin levels compared to controls (8.09 vs. 4.45 ng/mL, *p* = 0.06). When participants with a history of cardiovascular disease were excluded from the analysis and adjusting for multiple confounders through a regression model, the obesity group had higher presepsin values than the overweight and control groups (5.84 vs. 3.32 ng/mL, *p* = 0.016). In contrast, the overweight group had lower concentrations than both the obesity group (*p* = 0.005) and the controls (*p* = 0.031). We did not find an association between presepsin and 25-hydroxy vitamin D levels (*p* = 0.368). **Conclusions**: Although the cross-sectional character of the study cannot demonstrate causal relationships, the results could potentially suggest that systemic inflammation is implicated in the pathogenesis of obesity through the disruption of the intestinal barrier. However, the findings should only be seen as hypothesis-generating. The reduction in presepsin in the overweight state is an interesting finding that deserves further investigation.

## 1. Introduction

Obesity is increasingly recognized as a chronic relapsing disease that can cause serious complications and is associated with an increased risk of disability and mortality, poor quality of life, and significant economic cost to societies and health care systems [1]. Obesity is a multifactorial disorder with genetic, biological, social, behavioral, and cultural factors contributing to its development. Accumulating evidence suggests that intestinal dysbiosis is one of the key mechanisms involved in the pathogenesis of obesity and associated comorbidities through various mechanisms, including altered regulation of appetite and energy metabolism, increased lipogenesis and gluconeogenesis, and exacerbation of inflammation [2]. Furthermore, specific gut microbiota-derived metabolites, such as short-chain fatty acids and bile acids, have been associated with the risk of developing cardiovascular disease (CVD) in people living with obesity [3].

Previous studies have shown that metabolic disorders, including type 2 diabetes (T2D) and abdominal adiposity, are characterized by elevated circulating concentrations of Gram-negative bacteria lipopolysaccharide (LPS), even in the absence of infection, a phenomenon known as metabolic endotoxemia [4,5]. An imbalance in the gut bacteria population disrupts the intestinal barrier, leading to the penetration of LPS into the systemic circulation. Binding LPS to cell surface receptors activates pro-inflammatory signaling cascades, aggravating the vicious cycle of inflammation, which is a pathophysiological landmark of metabolic diseases and their complications [6]. Presepsin is the soluble fragment of an anchored glycoprotein called CD14, which serves as an LPS receptor and is involved in the generation of intracellular signals that regulate the host’s immune response to pathogens [7]. Although presepsin is detectable in healthy subjects at low concentrations, its levels increase rapidly in sepsis, presenting greater sensitivity and diagnostic accuracy for bacterial infections compared to conventional inflammation markers, such as procalcitonin and C-reactive protein (CRP) [8].

People with obesity are known to have low vitamin D levels for various reasons, such as greater volume distribution, tight bounding in adipose tissue, impaired absorption, and dietary habits [9]. Vitamin D is believed to exert potent anti-inflammatory properties through regulation of the expression of genes involved in the immune response and modulation of the synthesis of pro-inflammatory cytokines [10]. Previous studies have shown that vitamin D supplementation in children with overweight and obesity significantly improves inflammatory status, as reflected in a reduction in CRP levels [11]. Furthermore, vitamin D administration appears to positively affect the intestinal microbial signature in healthy individuals [12]. However, the link between presepsin and vitamin D has not been investigated so far.

In context with the above, we assumed that elevated levels of LPS in people with obesity should be accompanied by a respective increase in its ligand, that is, presepsin. However, to date, no data on presepsin levels are available in infection-free subjects (i.e., those who did not suffer from acute or chronic infection at the time of the investigation) with obesity or overweight. To better understand the role of gut dysbiosis and inflammation in obesity and in pursuit of novel biomarkers that correlate with the pathophysiological background of the disease, this study compared presepsin levels between three groups: people with obesity, overweight, and lean controls. A secondary objective was to investigate a possible relationship between presepsin levels and vitamin D status in people with overweight and obesity.

## 2. Materials and Methods

### 2.1. Inclusion and Exclusion Criteria

This cross-sectional study was carried out at the Aristotle University of Thessaloniki, Greece, between January and May 2024. The study included 81 participants (61.7% women): 27 in the obesity group, 34 in the overweight group, and 20 controls with normal body mass index (BMI). Participants in the obesity and overweight groups were recruited from the obesity clinics of the Hippokration and AHEPA university hospitals, while the control group consisted of hospital employees. The inclusion criteria were as follows: i. age over 18 years, ii. complete medical history at the time of inclusion in the study, and iii. BMI 18.5–24.9 kg/m^2^ for the control group, 25–29.9 kg/m^2^ for the overweight group, and ≥30 kg/m^2^ for the obesity group [13].

Exclusion criteria were the following: i. estimated glomerular filtration rate <60 mL/min/1.73 m^2^ since presepsin undergoes renal excretion; therefore, in people with kidney dysfunction, its levels are expected to be abnormally high [14], ii. age over 70 years, given that presepsin levels have been shown to increase in people older than 70 years compared to younger subjects [15], iii. acute or recent infections, surgical operations, or other severe inflammatory disorders (e.g., pancreatitis, burns, etc.), iv. history of diabetes mellitus or prediabetes, diagnosed according to the criteria of the American Diabetes Association [16], v. treatment with drugs that can affect body weight, such as corticosteroids, antipsychotics, etc., vi. severe liver disorder, and vii. history of autoimmune diseases and/or malignancies. The selection of inclusion and exclusion criteria was made with the aim of eliminating, as far as possible, confounding factors that could interact with presepsin levels in the study population.

Based on their BMI, study participants were classified into obesity, overweight, or control (normal weight) groups. Based on serum 25-hydroxy vitamin D [25(OH)D] levels, the vitamin D status was classified as deficient (<20 ng/mL), insufficient (21–29 ng/mL) or sufficient (≥30 ng/mL), according to the Clinical Practice Guidelines of the Endocrine Society [17]. To counteract any potential confounding effects of CVD and pharmacotherapy (e.g., statins) on inflammatory status, we also performed a secondary analysis in which participants with a history of CVD (defined as stroke, coronary artery disease, heart failure, or peripheral artery disease) were excluded.

### 2.2. Data Collection

Demographic, anthropometric, and laboratory parameters were recorded for each participant, all on the same day, to avoid seasonal variation in 25(OH)D concentrations. Demographic parameters included sex, age, and smoking status. The anthropometric parameters included body weight and height. Height was measured with a Holtain wall stadiometer. Body weight was recorded to the nearest 0.1 kg using a calibrated computerized digital balance (K-Tron P1-SR, Onrion LLC, Bergenfield, NJ, USA). Each participant was barefoot and dressed lightly during the assessment. The BMI was calculated by dividing the weight of a participant in kilograms by their height in meters squared. A second investigator confirmed the anthropometric evaluation separately.

### 2.3. Biochemical Analysis

Blood samples were drawn in the morning after a 12 h overnight fast by antecubital venipuncture, and serum samples were stored at −20 °C before analysis. 25(OH)D was assayed in the COBAS 8000 (e801) immunochemistry module using electrochemiluminescence technology (Roche Diagnostics, Rotkreuz, Switzerland). The reference ranges of values, as well as the inter- and intra-assay coefficients of variation for 25(OH)D, were as follows: ≥30 ng/mL, 2.2–6.8%, and 3.4–13.1%, respectively. Presepsin was determined by a sandwich enzyme-linked immune sorbent assay (ELISA) method (FineTest Human Presepsin ELISA kit, Wuhan Fine Biotech Co., Ltd., Wuhan, China). Anti-presepsin antibodies and biotin-conjugated detection anti-presepsin antibodies were used. Horseradish peroxidase (HRP)-streptavidin and 3,3′,5,5′-Tetramethylbenzidine (TMB) substrate were added to visualize the HRP enzymatic reaction. The absorbance was read at 450 nm in a microplate reader. The concentration of presepsin in the serum was calculated by drawing a standard curve. The concentration was proportional to the OD450 value. The Inter-Assay CV was <10% and the Intra-Assay CV was <8%.

### 2.4. Statistical Analysis

Frequencies and percentages were used to describe categorical variables in the study, and means with standard deviations to describe scale measurements. After normality testing, statistical inference for presepsin levels in the three groups of interest was based on medians and ranges, and comparisons were made using the Kruskal–Wallis test, while adjustments were made for multiple comparisons. Spearman’s Rho correlation coefficient was used to assess the statistical significance between presepsin levels and 25(OH)D, duration of intake, and age. Associations of obesity and vitamin D sufficiency or intake were examined using Pearson’s Chi-square test. A linear regression model was applied to assess independent prognostic factors for presepsin levels between the obesity and non-obesity groups after adjusting for the effects of smoking, age, sex, 25(OH)D levels, and vitamin D intake. Statistical significance was established at 0.05 in all cases, and the analysis was performed using SPSS v 26.0. 

### 2.5. Ethical Considerations

The study was carried out according to the principles of the Declaration of Helsinki and its subsequent amendments. All study participants gave their informed written consent before enrollment in the study, and the study protocol was approved by the Bioethics Committee of the Aristotle University of Thessaloniki (approval number 192/5-6-2020).

## 3. Results

### 3.1. Characteristics of the Study Population

**Figure 1:**
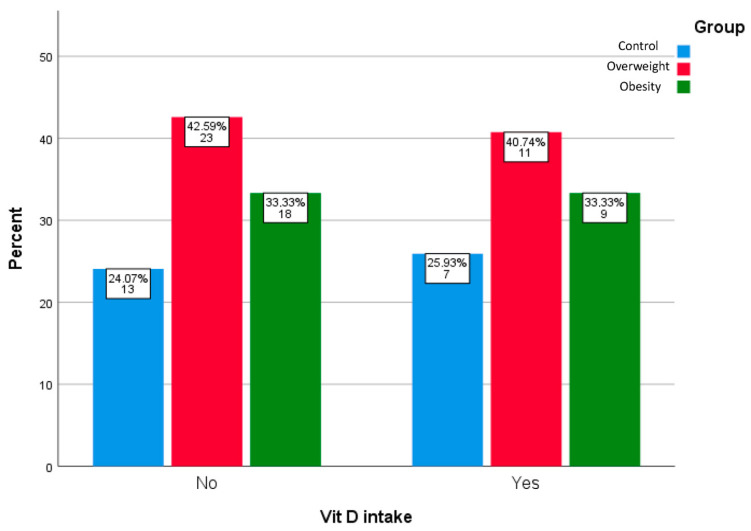
Percentages of vitamin D-supplemented and non-supplemented participants in the study groups.

The mean age, body weight, and BMI of the study sample were 47.48 years, 81.63 kg, and 28.81 kg/m^2^, respectively. Among study subjects, 33.3% were supplemented with vitamin D (prescribed by a primary care physician or pharmacist or consumed by participants over the counter). As expected, the obesity, overweight, and control groups differed significantly in terms of BMI (35.18 vs. 27.45 vs. 22.53 kg/m^2^, respectively, *p* < 0.01) and body weight (98.07 vs. 78.50 vs. 64.75 kg, respectively, *p* < 0.01). In contrast, there were no significant differences between the groups regarding mean age (47.44 vs. 49.88 vs. 43.45 years, respectively), sex distribution (55.55 vs. 55.88 vs. 75% women, respectively), smoking status (25.92 vs. 35.29 vs. 40%, respectively), CVD history (18.51 vs. 23.52 vs. 10%, respectively), vitamin D sufficiency status (18.18 vs. 40.91 vs. 40.91%, respectively) and percentage of supplemented participants (33.33 vs. 40.74% vs. 25.93%, respectively) (Figure 1); *p* > 0.05 in all cases. Mean 25(OH)D levels (24.24 vs. 25.63 vs. 28.19 ng/mL) were also similar across the groups (*p* = 0.179).

Table 1 presents the characteristics of the study population, classified into BMI groups.

### 3.2. Presepsin Levels in Study Groups

**Figure 2:**
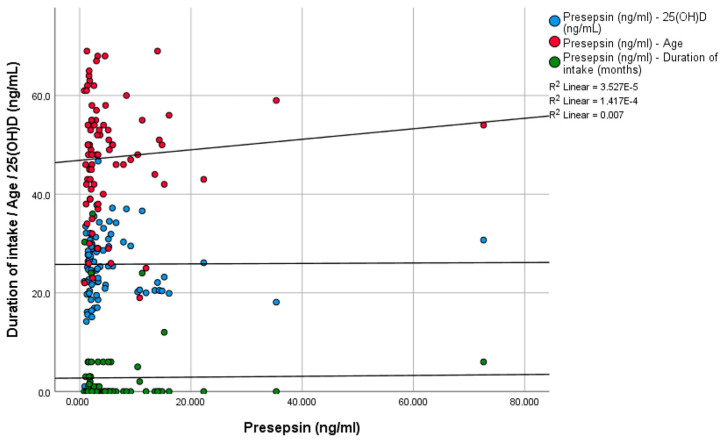
Relationship between presepsin and age, 25(OH)D levels, and duration of vitamin D intake.

When the sum of study participants was included in the analysis, presepsin was found to be almost twice higher in the obesity group than in the control group; however, the difference was found to be marginally non-significant (8.09 vs. 4.45 ng/mL, *p* = 0.067). When 15 participants with a history of CVD were excluded from the analysis, presepsin concentrations were found to be significantly higher in the obesity group compared to the sum of participants in the overweight and control groups (*p* = 0.033). Furthermore, the overweight group had significantly lower presepsin levels compared to both the control group (*p* = 0.031) and the obesity group (*p* = 0.005). We did not find a significant relationship between presepsin values and age (*p* = 0.892), 25(OH)D levels (*p* = 0.368), duration of supplementation (*p* = 0.587), and vitamin D status (*p* = 0.692) (Figure 2).

The regression model indicated higher presepsin levels in the obesity group compared with the overweight and control groups (5.84 vs. 3.32 ng/mL, β = 1.271; *p* = 0.016), while adjusting for age (*p* = 0.954), sex (*p* = 0.951), 25(OH)D levels (*p* = 0.908), smoking status (*p* = 0.832), and vitamin D intake (*p* = 0.792) (Table 2).

**Figure 3:**
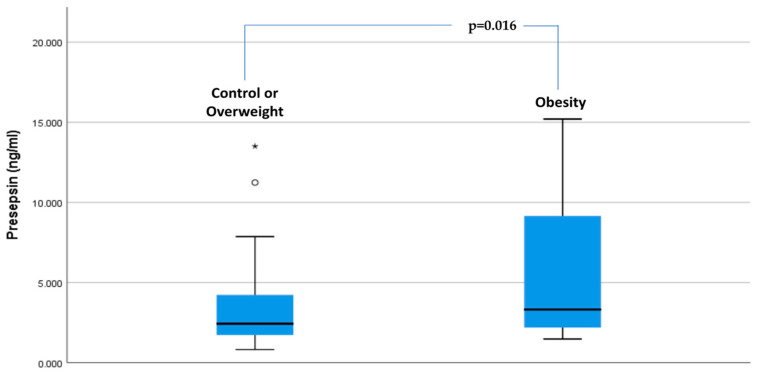
Presepsin concentrations in the study groups. ° Outliers * Extreme outliers.

Figure 3 illustrates presepsin concentrations across the study groups after the exclusion of participants with a history of CVD.

## 4. Discussion

To the best of our knowledge, this is the first study in the literature in which presepsin, a biomarker previously exclusively associated with sepsis, was evaluated in infection-free subjects with overweight and obesity. Our findings demonstrate an increase in presepsin concentrations in people living with obesity compared to controls of normal weight. Additionally, we observed a rather unexpected reduction in presepsin in the overweight state, while we did not find a significant relationship between presepsin values and 25(OH)D levels.

It is well established that obesity is a state of low-grade inflammation. In a large cross-sectional study that included more than 16,000 subjects, Visser et al. [18] showed that a higher BMI is associated with higher CRP concentrations after adjustment for several confounders, including smoking and health status. Abbasi et al. [19] demonstrated that procalcitonin, another well-known biomarker of sepsis and bacterial infections, is positively associated with BMI and markers of insulin resistance. Unlike our findings, both CRP and procalcitonin appear to increase in parallel with BMI, suggesting a gradual aggravation of inflammation as BMI values move from the lean to the obese state. Although it is difficult to provide an explanation for the decrease in presepsin concentrations that we observed in the overweight group, it could be assumed that it represents a counterregulatory mechanism to the respective increase in circulating LPS levels. For some reasons that deserve further investigation, this protective adaptation wanes once a weight or adiposity threshold is reached, leading to the solidification of increased presepsin concentrations in obesity.

Although presepsin has traditionally been considered a biomarker of sepsis, preliminary evidence suggests that it can accurately reflect metabolic dysregulation, reinforcing the notion that inflammation, but also immune responses, are involved in the pathogenesis of cardiometabolic disorders [20]. In a cross-sectional study, Kouroupis et al. [21] showed that presepsin levels are lower in infection-free people with well-controlled T2D compared to peers with adequately controlled type 1 diabetes (T1D) and are associated with the duration of diabetes. These data suggest that drugs used in the management of T2D, such as antidiabetics and statins, can reduce presepsin through their actions in the intestinal tract and their effects on the microbiome [22]. Based on these findings and to counteract any potential effects of pharmacotherapy on presepsin levels, we performed a secondary analysis in which CVD patients were excluded, including only participants with obesity but without chronic use of medications. The findings of the secondary analysis were in agreement with those of the primary analysis, confirming the higher presepsin concentrations in the obesity group than in the comparison groups. Another study that included only infection-free patients with T1D using continuous glucose monitoring (CGM) showed that presepsin values correlate with CGM metrics [23]. These data are in line with the findings of the present study and suggest a potential role for presepsin in the monitoring of metabolic disorders that extends beyond its clinical utility in sepsis.

Using data from the English Longitudinal Study of Aging (ELSA), de Oliveira et al. [24] showed that low vitamin D concentrations are associated with increased levels of inflammation markers, including CRP and white blood cell count. Other studies have also established a significant association between 25(OH)D concentrations and levels of interleukins 6 (IL-6) and 10 [25]. However, it should be mentioned that both of the above studies included elderly individuals. Aging is associated with persistent activation of the immune system and a high level of circulating inflammatory markers [26]. The relatively young mean age of our study population, in conjunction with the small sample size, could explain the discordance between the findings of the present study and those of the previous ones. However, given that vitamin D treatment has been shown to positively affect the gut microbiota and alleviate inflammatory changes in the colon epithelium in a mouse model of LPS-stimulated systemic inflammation [27], the relationship between presepsin and vitamin D deserves further evaluation in larger studies.

The strength of the present study lies in its novelty and the fact that the three groups were carefully balanced in terms of age, sex distribution, smoking status, history of CVD, and 25(OH)D levels, while statistical adjustments for the above confounders confirmed the consistency of our observations. On the other hand, its findings should be interpreted in light of important limitations. The cross-sectional design cannot prove causal relationships, while the small sample size and pilot nature could have limited the potential of the study to reveal significant associations. Given the pilot character of the study, we did not proceed with a post hoc power analysis, which might have been misleading and not informative for data interpretation [28]. Furthermore, although BMI currently remains the metric of choice for the diagnosis and classification of obesity, it cannot adequately reflect the degree of adiposity that is the main driver of obesity-related complications. Evaluating the dietary habits of participants that are known to affect the intestinal flora [29] would provide a deeper understanding of presepsin alterations in overweight and obesity. Finally, the claim of a possible association between presepsin and obesity could not be fully substantiated without the parallel evaluation of other biomarkers that are part of the same biological circuit. For example, simultaneous measurement of presepsin and LPS levels could confirm or reject the hypothesis of a parallel change in the two markers in obesity as a consequence of intestinal dysbiosis. Ikegame et al. [30] have shown that presepsin production is influenced by monocyte phagocytosis of neutrophil extracellular traps induced by CD14 expression. Toprak et al. [31] recently demonstrated that presepsin and zonulin, a protein that modulates the permeability of narrow junctions between cells of the intestinal tract wall, may be useful therapeutic and diagnostic targets for acute myocarditis. In a study that aimed to characterize the kinetics of presepsin and associated inflammatory markers in healthy subjects with experimentally induced endotoxemia, Aulin et al. [32] found direct interactions between presepsin, tumor necrosis factor-α and various interleukins. These interesting data indicate that CD14, zonulin, and cytokines are biomarkers that could be investigated in conjunction with presepsin in infection-free individuals with obesity to provide a clearer picture of the complex underlying pathophysiology. For these reasons, it is important to emphasize that the results of the present study should be considered exploratory.

In conclusion, the findings of this pilot study should only be seen as hypothesis-generating; however, they could potentially contribute to the existing knowledge that intestinal dysbiosis is implicated in the pathophysiology of obesity by aggravating systemic inflammation. The role of presepsin as a biomarker of obesity and its complications, as well as the reduction in presepsin in the overweight state, warrant further investigation in future studies with a larger sample size, a deeper mechanistic approach, and a prospective design.

## Figures and Tables

**Table 1:** Characteristics of the study population are classified into groups based on BMI.

		Group								
		Control (*n* = 20)		Overweight (*n* = 34)			Obesity (*n* = 27)			
	Mean	SD	Median	Mean	SD	Median	Mean	SD	Median	*p*-Value
Age (years)	43.45	11.98	46	49.88	13.01	50	47.44	10.09	50	0.542
Weight (kg)	64.75	8.88	64	78.50	11.22	75	98.07	21.79	90	<0.01
Height (m)	1.69	0.09	1.67	1.69	0.12	1.67	1.67	0.10	1.67	0.752
BMI (kg/m^2^)	22.53	1.19	22.9	27.45	1.31	27.5	35.18	5.86	33.4	<0.01
Duration of intake (months)	6.07	10.80	0.5	1.90	5.74	0.0	1.44	2.98	0.0	0.324
25(OH)D (ng/mL)	28.19	9.21	29.2	25.63	7.02	25.3	24.24	5.28	23.2	0.179
Presepsin (ng/mL)	4.45	3.24	3.30	4.01	6.94	2.16	8.09	13.79	3.80	0.067

BMI: body mass index; SD: standard deviation; 25(OH)D: 25-hydroxy-vitamin D.

**Table 2:** Results of the linear regression analysis.

Parameter	BETA	95% Lower Bound	95%Upper Bound	*p*-Value
Constant	2.150	−4.621	8.922	0.527
Group	1.271	0.224	2.323	0.016
Age	−0.003	−0.091	0.086	0.954
Sex	−0.068	−2.281	2.146	0.951
25(OH)D	0.008	−0.129	0.144	0.908
Vitamin D intake	−0.293	−2.502	1.917	0.792
Smoking	0.321	−2.131	1.453	0.832

25(OH)D: 25-hydroxy-vitamin D.

## Data Availability

The data presented in the study are available on request from the corresponding author. The data are not publicly available due to privacy restrictions of the Greek National Health System.
